# Foraging dive frequency predicts body mass gain in the Adélie penguin

**DOI:** 10.1038/s41598-021-02451-4

**Published:** 2021-11-24

**Authors:** Amélie Lescroël, Annie Schmidt, Megan Elrod, David G. Ainley, Grant Ballard

**Affiliations:** 1 Point Blue Conservation Science, Petaluma, CA 94954 USA; 2 H. T. Harvey & Associates Ecological Consultants, Los Gatos, CA 95032 USA

**Keywords:** Ecology, Behavioural ecology, Ocean sciences

## Abstract

Quantifying food intake in wild animals is crucial to many ecological and evolutionary questions, yet it can be very challenging, especially in the marine environment. Because foraging behavior can be inferred from dive recordings in many marine creatures, we hypothesized that specific behavioral dive variables can indicate food intake. To test this hypothesis, we attached time-depth recorders to breeding Adélie penguins also implanted with RFID tags that crossed a weighbridge as they traveled to and from the ocean to feed their chicks. The weighbridge reported how much mass the penguin had gained during a foraging trip. The variables that explained a significant amount of the change in body mass while at sea were the number of foraging dives per hour (46%) and the number of undulations per hour (12%). Most importantly, every increment of 1 in the rate of foraging dives per hour equated to a penguin gaining an average 170 g of mass, over the course of a 6–60 h foraging trip. These results add to a growing understanding that different metrics of foraging success are likely appropriate for different species, and that assessing the types and frequencies of dives using time-depth recorders can yield valuable insights.

## Introduction

Understanding ‘how organisms acquire and then make use of resources in metabolism, movement, growth, reproduction, and so on’^1^ is the foundation of functional ecology. Quantifying how much food, and ultimately energy, animals are able to collect, and where, is crucial to answering many ecological and evolutionary questions, as well as to conservation efforts. For example, quantifying the temporal, spatial, and inter-individual variability in food intake is essential to understand how environmental variation can affect population processes^2^, how some individuals consistently achieve higher breeding performance than others^3^, or how individual characteristics such as age, sex, and experience can influence foraging performance^4,5^. Determining food intake also plays a crucial role in testing fundamental theories in ecology (e.g. optimal foraging theory^6^), determining habitat use and important foraging grounds for vulnerable species^7^ or inferring prey distribution in remote environments^8^.

However, quantifying food intake can be challenging in wild populations. It is especially difficult in the marine environment where, most of the time, humans are at a disadvantage in not being able to directly observe individual animals that often catch their prey underwater. Over the past 30 years, advances in bio-logging technology (i.e. the use of animal-borne units to collect data) has allowed tremendous progress in our understanding of the physiology, behavior, ecology and social interactions of wild marine animals^9–11^. Recently, the use of miniaturized accelerometers (accurately measuring the body or body part movements in 2–3 axes) together with onboard video cameras^12,13^ finally allowed quantifying prey capture at the scale of a single dive in seabirds. While an indisputable advance, these recent studies involved equipping individuals with 2–3 different devices that had to be recovered so that the large amount of high-definition data could be analyzed through frame-by-frame video screening and complex signal analysis. For now, this is one of the few ways (see also the use of Hall sensors and magnet on penguin bills^14^, and of oesophagus or stomach temperature sensors^15–17^) to relate a specific diving event, in time and space, to the capture of a specific prey. However, this is only feasible in the context of short-term deployments during the breeding season.

Early-life (juvenile phase) and winter ecology are the next frontiers to explore to expand our understanding of the relationships between marine animals and their environment. These investigations require long-term data collection (over the course of 8–9 months for winter ecology, up to 2–5 years for early-life ecology), hence the use of the least cumbersome devices possible, and/or a data transmission system via satellites. Attaching devices to marine creatures, especially through increased drag, can easily compromise their movements and foraging^18^. This precludes, for now, the use of video and high-resolution acceleration data, because the devices are still too large to be deployed for long-durations and the data are too extensive to be transmitted or stored over long periods. However the interpretation of indirect data, such as two-dimensional (2D) diving profiles, from which simple variables can be computed and summarized^8,19^, still holds promise. In diving birds and mammals, numerous dive metrics (e.g. undulations in the dive profile, dive depth and duration, bottom duration, dive bottom duration, descent and ascent rates, number of dives) have been used as indexes of prey acquisition^19–23^, although results of different studies have often been inconsistent, species-specific, or location-specific, calling for validation in each study system.

The number of undulations has been shown to be linearly related to the number of prey captures for both fish- and krill-eating penguins feeding in the water column such as Adélie penguins (but not yet for benthic feeders^16,24,25^), although it was established that this proxy could not be used to quantify the exact number of prey captures, as a small amount of undulations could occur without prey capture and vice-versa^12,25^. In Magellanic penguins (*Spheniscus magellanicus*), any single undulation had an 86% chance of being associated with a prey capture, but two adjacent undulations increased the probability of at least one prey being taken to 98%, indicating that several undulations per dive was a better indication of prey catch^24^. Similarly, it has been reported that the success of Adélie penguins feeding on krill during a foraging trip depended on a relatively small number of very successful dives rather than the total number of dives^12^, hence explaining why time spent diving by breeding Adélie penguins does not affect brood growth rates^26^. In common guillemots (*Uria aalge*) and razorbills (*Alca torda*), dive duration and depth were good predictors of prey catch events^8^, and dive bottom duration showed a significant positive linear relationship with number of underwater beak-opening events (indicating feeding) during dives in chinstrap penguins (*Pygoscelis antarctica*)^25^. These feeding events were also more frequent during deeper dives. In thick-billed murres (*Uria lomvia*), it is the number of dives (as well as flight time) that correlated to the mass of prey brought back to the nest^21^. In Antarctic fur seals (*Arctocephalus gazella*), the number of prey capture attempts was best predicted by descent and ascent rates at the dive scale and interestingly, in this species which appears to pursue its prey from above contrary to penguins, the number of undulations was unrelated to foraging success^19^. These diverse results call for the need to specifically assess the validity and strength of potential indexes of foraging success for each study species^19^, and to include more than one dive metric when inferring foraging success from dive data only. The Adélie penguin (*Pygoscelis adeliae*) is one of the best studied seabird species, yet measuring prey acquisition of Adélie penguins at a relatively fine temporal scale over extended periods is still a challenge (cf.^12^ for short-term periods). Undulations or “wiggles” (i.e. changes in vertical direction) in the dive profile have been linked to drops in oesophagal temperature thought to reflect prey capture events^16^ and used subsequently, but without additional validation, as an index of foraging success^4,5,22,23,27^. In a few Adélie penguin colonies and other penguin species colonies, radio-frequency identification (RFID) systems coupled with automated weighbridges ( i.e. scales) have been installed since the 1990s to monitor trip duration, frequency, body mass changes or survival of RFID-implanted penguins^28–31^. Used in conjunction with time-depth recorders, these systems can tell us how much mass has been gained (or lost) by an individual penguin after a given foraging trip at sea and this information can be matched to the corresponding 2D diving profile.

In the present study, we used small, leg-mounted time-depth recorders attached to RFID-implanted birds to test the hypothesis that specific behavioral dive variables can provide a reliable measure of food intake in free-ranging marine predators. These behavioral variables could subsequently be used to identify areas and time periods of high foraging intensity outside of the breeding season.

## Material and methods

### Study site and system

Data were collected at Cape Crozier (77°27′S, 169°12′E), Ross Island, one of the largest Adélie penguin breeding colonies (~ 275 000 pairs at the time of the study^32^), during austral summer 2018–2019. Individuals arrive at Cape Crozier in late October/early November, lay (usually two) eggs in mid-November, and feed their chicks between mid-December and early February. They are one of the few penguin species that can fledge two chicks. During the brood/guard stage, one parent remains with the chick(s) while the other forages at sea. Nest reliefs at Crozier occur every 1–2 days during early chick-rearing and chicks are fed relatively small meals (0.43–0.58 kg) by the attending parent^33^. After about two weeks, chick demands are too great for adequate provisioning by one parent, so chicks are left on their own (“crèche” stage) while both parents forage simultaneously. Our study period included most of chick-rearing, i.e., all of the guard stage and half the crèche stage, from December 21, 2018 to January 15, 2019.

Since 1997, every austral summer, the same subcolony of ~ 200 pairs (152 pairs in the year of study) was surrounded by a plastic fence, leaving only one opening as an access point, where the weighbridge was located^30^. The weighbridge consisted of an electronic scale, direction indicator, and radio frequency identification (RFID) reader^34,35^. In 2018–2019, it was installed on November 16 and removed on January 20. A subset of adult individuals were implanted with unique RFID tags beginning in 1997, with a few more added each year^30,36^. RFID code, date and time, direction, and weight were recorded automatically as the RFID-implanted birds crossed the weighbridge. Adults were captured on the nest during incubation, when they can be approached slowly and gently lifted off their nest. A warm hat was placed over the eggs or small chicks to avoid chilling, while the RFID tag was injected into the bird.

All penguin survey, capture and handling methods used for data collection were performed following all relevant guidelines and regulations under the approval and oversight of the Institutional Animal Care and Use Committees of Oregon State University and Point Blue Conservation Science. Additionally, all work was approved and conducted under Antarctic Conservation Act permits issued by the US National Science Foundation and the U.S. Antarctic Program. The study is reported in accordance with ARRIVE guidelines.

### Diving parameters

**Figure 1:**
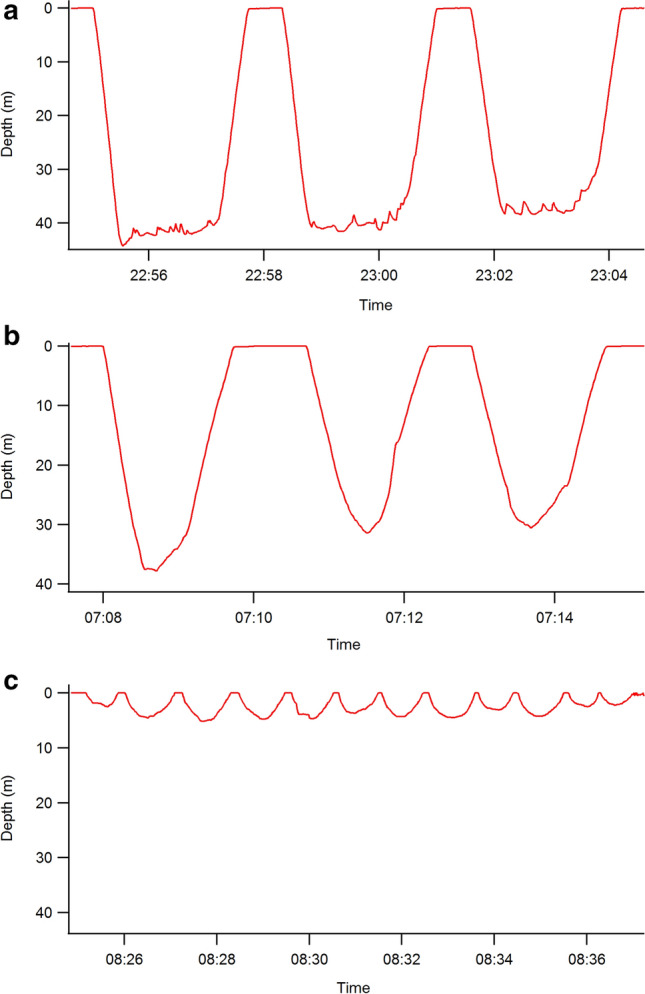
Adélie penguin dives were classified into ‘foraging’, ‘exploratory’ and ‘other’^39^. (**a**) foraging dives were at least 10 m deep, had significant bottom time and many undulations (cases a–c in the methods); (**b**) exploratory dives were at least 10 m deep but had relatively little bottom time and/or few undulations (cases d, e in the methods); (**c**) other dives, thought to be primarily commuting dives, were shallower than 10 m and/or not matching the requirements for foraging and exploratory dives.

Between November 2 and December 7, 2018, we equipped 32 RFID-implanted birds with geolocating dive recorders (“LUL” tags, 22 × 21 × 15 mm, weight = 4 g, from Atesys, Strasbourg, France, hereafter referred to as GDRs) that recorded light every minute, temperature (with a precision of ± 0.5 °C) every 30 s and pressure (with a precision of ± 0.3 m) every second for 12–15 months. Adults were captured using a hand net (2 m long handle) or on the nest during incubation (see above). The GDRs were encapsulated in flexible heat-shrink tubing shaped into a leg strap and attached to the tibio-fibula of each bird in the field using a polyester-coated stainless-steel zip tie to secure the ends of the strap together such that the tag could rotate freely around the leg but not slip over the tarsus joint. Tags were left in place for one year, with 21 recovered at the beginning of the 2019–2020 breeding season. Pressure data were processed in R (v. 3.6.0) with several processes modified from the diveMove package (v. 1.4.5)^37^. To correct for instrument drift, pressure data were zero offset corrected using the calibrateDepth function^38^. We used a depth threshold of 3 m to qualify as a dive. Following methods described in previous studies^27,39,40^, we computed a number of statistics about each dive including dive duration, maximum dive depth, post-dive interval duration, bottom time, the number of undulations (changes of any amplitude in underwater swimming duration from either ascent to descent, or descent to ascent—used for the purposes of categorizing dives) and the number of undulations > 1 m (changes in underwater swimming direction from ascent to descent > 1m^39^). The two undulation metrics are highly correlated (Pearson’s r = 0.92 in our data set). Bottom time was defined as the time spent at > 60% of maximum depth of dive with < 0.5 m/s change in depth. Based on these statistics, dives were classified into ‘foraging’, ‘exploratory’ and ‘other’^39^ (Fig. 1). Foraging and exploratory dives both were at least 10 m. To be classified as a foraging dive, a dive needed to have one of the following set of characteristics: (a) bottom time of at least 20 s, max depth of at least 15 m and more than 4 undulations, (b) bottom time of at least 15 s, max depth of at least 10 m, total dive time of at least 30 s, more than 4 undulations, at least 30% of the dive duration spent in slow depth change rate and 30% with fast depth change rate, (c) bottom time of at least 15 s, max depth of at least 10 m, total dive time of at least 30 s, more than 6 undulations and very fast (at least 1 m s^−1^) ascent/descent phases. To be classified as an exploratory dive, a dive needed to have one of the following set of characteristics: (d) max depth of at least 15 m and either less than 20 s of bottom time or less than 4 undulations, (e) max depth of at least 10 m, less than 15 s of bottom time, less than 6 undulations and fast (at least 0.8 m s^−1^) ascent/descent phases. All other dives were categorized as ‘other’ and are thought to be primarily shallow commuting dives^41^.

### Foraging trip estimation and duration

**Figure 2:**
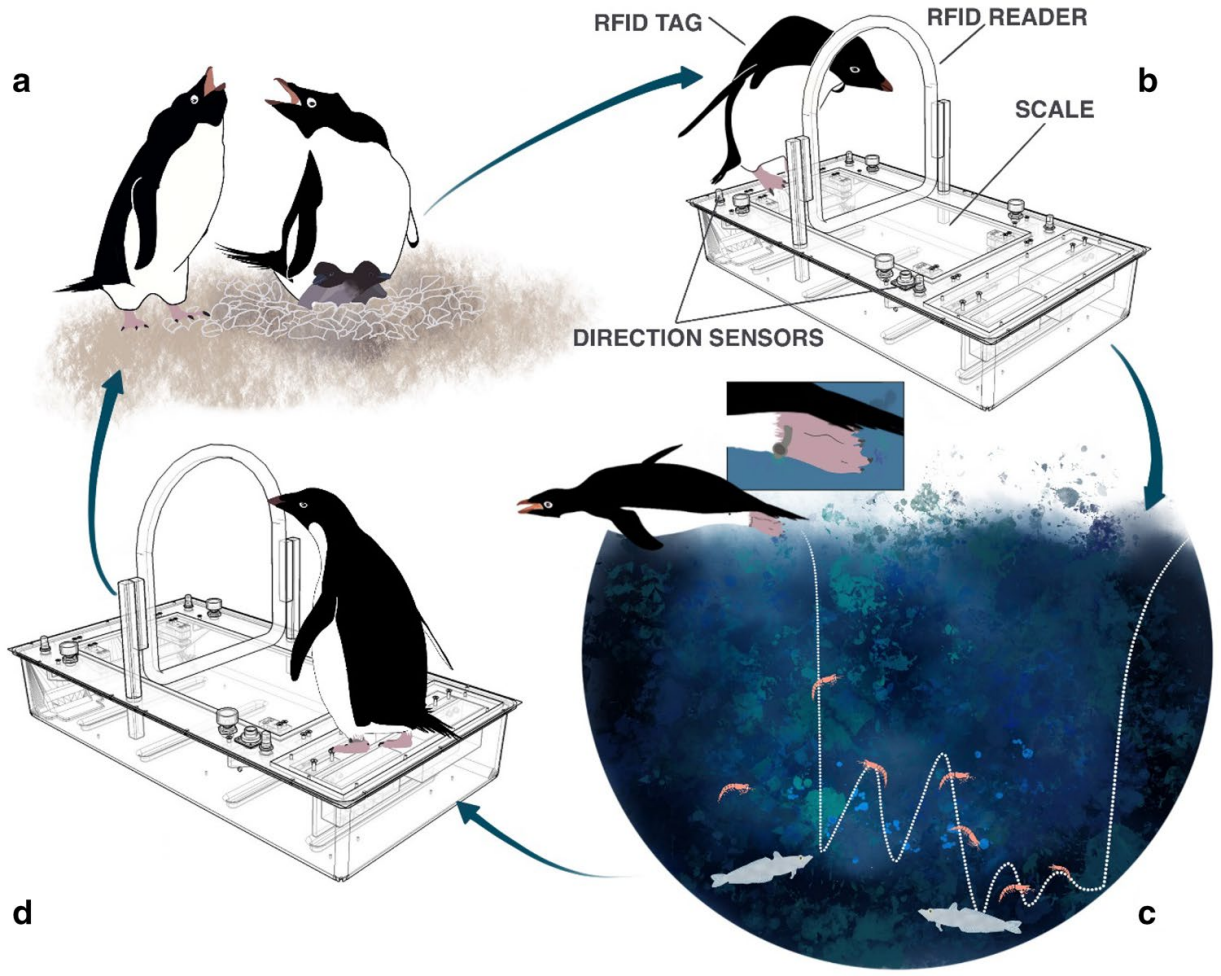
Conceptual visualization of the study design. (**a**) chick-rearing Adélie penguins breeding in a semi-enclosed subcolony are implanted with a RFID tag and equipped with a leg-mounted time-depth recorder (GDR). (**b**) Bird ID, departure mass and direction of travel are recorded by the weighbridge as penguins leave the colony to forage at sea. (**c**) During the foraging trip, the GDR tag records depth every second, enabling the calculation of several dive behavior metrics. (**d**) Bird ID, return mass and direction of travel are recorded by the weighbridge as penguins return to the colony to feed their chicks.

A foraging trip was defined as the duration elapsed between the exit over the WB of a RFID-implanted bird (after having first been recorded as entering the subcolony) and its subsequent recrossing upon return to the subcolony (Fig. 2). In order to avoid including non-foraging trips (i.e. for nest maintenance purposes), we excluded trips that were shorter than 6 hrs^36^. To minimize the occurrence of resting periods while outside of the subcolony, we selected foraging trips performed between December 21, 2018 and January 15, 2019, while the birds were actively provisioning chicks. To further reduce the influence of digestion on body mass changes over the trip^42^, and after the visual examination of the distribution of trip durations, we also excluded trips that were > 60 h (trip duration during chick-rearing takes 1–2 days on average^36,39^ but their frequency distribution showed a tail from 60 to 100 h in our data).

### Body mass estimation

For each foraging trip, we calculated meal size and body mass change (see Supplementary Information for more details on the weight calculation). Meal size (in kg) is the difference between an individual’s out-mass (departing) and its most recent in-mass (returning from sea), i.e. this is a measure of how much food a parent left in the colony and includes both the food delivered to chicks and the food digested by the parent while attending the nest^39^. Body mass change (in kg) of individual birds over each foraging trip was calculated as the return mass (post-foraging trip at sea) minus the departure mass (pre-foraging trip at sea). Hence, body mass change measures the amount of food that was collected during the trip at sea (i.e. foraging success^43^), minus what could have been digested before returning to the colony at the end of this trip (Fig. 2). We further filtered trips based on these two variables, keeping only trips where meal size was > 0 and < 1.3 kg^33^ and body mass change was > − 0.8 and < 1.6 kg (confirmed to be in the range of mass changes directly measured in the field using Pesola scales^44^). Our final data set included 25 foraging trips and associated body mass changes (i.e. 2.3 trips per bird, ranging 1–4 trips per bird) from 11 GDR-equipped birds (including 11 trips from 6 females and 14 trips from 5 males). Birds were sexed by DNA from a feather sample (n = 6), or lacking that, by a combination of size, behavior, and timing of colony attendance^45–47^ (n = 5). Data collected during a previous study (from 2010 to 2013, n = 140) showed that we were able to assign the correct sex to 97.14% of the birds using this latter method when compared with DNA data on the same birds^48^.

### Foraging success indexes

Based on the estimated dive parameters and on existing literature, we selected the following behavioral variables as potential indexes of food intake (all per hour metrics computed as the total over the entire foraging trip divided by the trip duration in hours): (1) number of undulations > 1 m per hour, as previous work indicated that undulations in the dive profile represent feeding and/or prey capture^16,24,25^, (2) dive (underwater) time per hour, (3) dive time per hour during foraging dives only, (4) bottom time per hour, (5) number of foraging dives per hour, (6) Attempts of Catch per Unit Effort (ACPUE, calculated as the number of undulations per trip divided by total bottom duration^23,49^). We also considered two variables calculated at the scale of dive bouts: (7) mean bout duration, thought to reflect the time spent within a prey patch^50,51^, (8) number of dives per bout, as an index of the size of the prey patch^51–53^. Dive bouts were defined as successive diving events interrupted by relatively longer surfacing periods. To separate post-dive intervals from inter-bout duration, we used a maximum likelihood approach^54^ using the diveMove package^37^ in R, which allowed us to determine a bout-ending-criterion (BEC). In this study, BEC = 47.6 s.

### Statistical analyses

We first calculated a Pearson correlation matrix using the corrplot package in R and removed highly correlated (r > 0.7) behavioral covariates, keeping those that were the most correlated with body mass change. To test the hypothesis that some behavioral dive variables can be used to predict the amount of food collected while foraging at sea, we evaluated linear mixed models including body mass change as the dependent variable, each of the selected behavioral variables as independent variables and bird ID as a random effect, as well as a null model (intercept only) using the nlme package^55^ in R. Once we had determined the most competitive models, and as Adélie penguin’s foraging success can vary according to sex^29,36^ and chick needs^39^, and also be influenced by the trip duration^56^, we added sex, study day (day in the season as a Julian date with Dec 20 = 0) and trip duration (in hours) to the top intrinsic model(s) including potential interactions with the selected behavioral variable(s). A null model was also included in this second model set. Residuals were examined to verify normality, homogeneity of variances, and independence. To evaluate these models and determine the strength of evidence supporting specific effects, we used an information theoretic approach^57^. Models were ranked using the small-sample-size corrected version of Akaike Information Criterion (AICc), with the best model having the lowest AICc value. We calculated ΔAICc as the difference in AICc between each candidate model and the model with the lowest AICc value, and considered all models within 2 ΔAICc as competitive models^57^. We determined the strength of evidence supporting specific effects by examining the unstandardized effect sizes (slope coefficients and differences in means) and the associated 95% confidence intervals (CI). If the 95% CI for a parameter in a competitive model (ΔAICc < 2.0) included zero, it was considered uninformative. Models were fitted using maximum likelihood (ML) estimation during model selection (in order to choose the best fixed effect structure), then the best model was fitted using restricted ML (REML) estimation to obtain accurate parameter estimates^58^.

To test for the effect of GDRs on trip duration and mass change, we used all available WB records for the season (including RFID-implanted birds with and without GDRs) filtered on dates, breeding status, trip duration and weights as detailed above (resulting in a data set of 122 trips, ranging 1–13 trips per individual). We then fitted linear mixed models using REML with either trip duration or mass change as the dependent variable, the presence/absence of GDR, sex and study day as independent variables and individual as a random effect.

All statistics from this section were performed using R 4.0.3. Means ± SE are given unless indicated otherwise.

## Results

**Figure 3:**
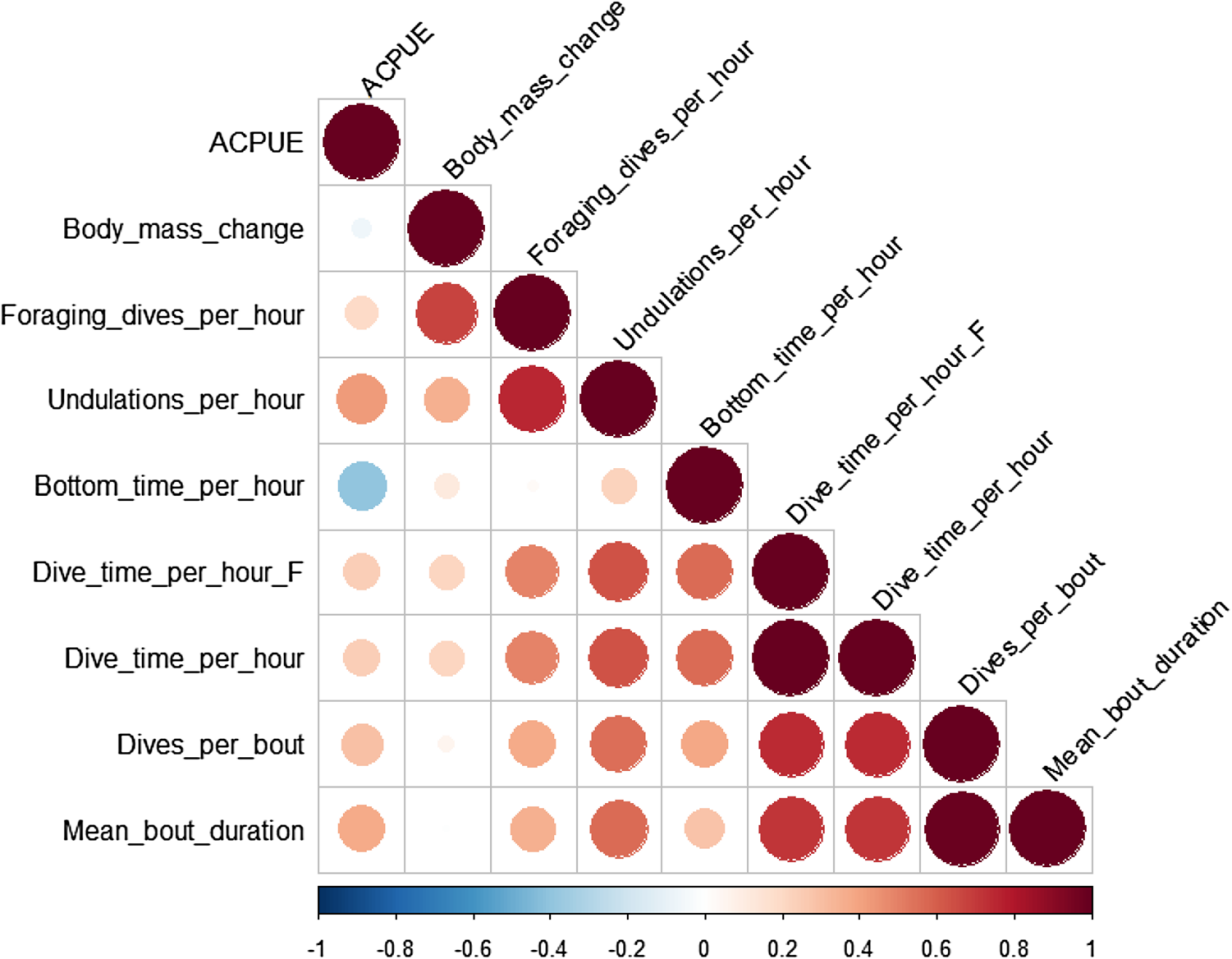
Correlation matrix (Pearson) showing the strength of the correlation between body mass change in chick-rearing Adélie penguins and behavioral variables (n = 25). Dive_time_per_hour: dive (underwater) time per hour, Dive_time_per_hour_F: dive time per hour during foraging dives only.

Across all individuals, body mass change among foraging trips varied from − 0.647 to 0.904 kg (mean ± SD = 0.181 ± 0.421 kg). The behavioral variables that were most correlated to body mass change were number of foraging dives per hour, number of undulations (> 1 m) per hour, dive time per hour (both all dives and foraging dives only), and bottom time per hour (Fig. 3). Both measures of dive time per hour (dive time per hour and dive time per hour during foraging dives only) were highly correlated with the mean bout duration and the number of dives per bout; only the dive time per hour during foraging dives was kept for the rest of analysis as it was more strongly correlated with body mass change. The number of undulations per hour was also highly correlated with the bottom time per hour and only the number of undulations per hour was kept as it was more strongly correlated with body mass change.

### Effect of devices on trip duration and mass change

Upon comparing RFID birds with GDR tags vs those without, and taking into account sex and study day, the presence of the GDR did not significantly affect trip duration ($\widehat{\beta }$ = 3.910, SE = 3.543, 95% CI − 3.269 to 11.0882) or mass change ($\widehat{\beta }$ = − 0.096, SE = 0.090, 95% CI − 0.279 to 0.087).

### Effect of foraging behavior on body mass change

The top model (Table 1, model 1) included a linear effect of the number of foraging dives per hour ($\widehat{\beta }$ = 0.170, SE = 0.039, 95% CI 0.085 to 0.255), explaining 46% of the variation in body mass change. According to this model, every increment of 1 in the rate of foraging dives per hour equated to a penguin gaining on average 170 ± 0.04 g of mass over the course of a 6–60 h foraging trip. The number of undulations per hour explained 12% of the variation in body mass change and the model including this variable received far less support than the top model (Table 1, model 2).

**Table 1:** Modelling the foraging success (in terms of body mass change over a foraging trip) according to behavioral (diving) variables (and other covariates) for breeding Adélie penguins at Cape Crozier, Ross Island, 2018/2019.

No	Model	ΔAICc	K	Deviance	R^2^
	**Body mass change ~ diving variables (n = 25)**				
1	**Foraging_dives_per_hour**	**0**	**4**	**11.785**	**0.46**
2	Undulations_per_hour	11.685	4	23.471	0.12
3	Null	11.620	3	26.662	0.00
4	Dive_time_per_hour_F	13.717	4	25.503	0.05
	**Body mass change ~ diving variables + /x other covariates (n = 25)**				
1	**Foraging_dives_per_hour**	**0**	**4**	**11.785**	**0.46**
6	**Foraging_dives_per_hour + Study_day**	**1.510**	**5**	**10.137**	**0.49**
7	**Foraging_dives_per_hour + Sex**	**1.988**	**5**	**10.616**	**0.48**
8	Foraging_dives_per_hour + Trip_duration	2.192	5	10.819	0.48
9	Foraging_dives_per_hour * Sex	3.985	6	9.103	0.52
10	Foraging_dives_per_hour * Study_day	4.928	6	10.046	0.50
11	Foraging_dives_per_hour * Trip_duration	5.423	6	10.542	0.49
12	Trip_duration	8.336	4	20.121	0.25
3	Null	12.019	3	26.662	0.00
13	Study_day	13.620	4	25.405	0.05
14	Sex	14.856	4	26.641	0.00

Models within 2 ΔAICc of the best model are in bold. Models were fitted using maximum likelihood estimation with bird ID as a random effect. R^2^ = marginal r-squared value for mixed models from Nakagawa et al.^59^.

### Effect of other covariates on body mass change

**Figure 4:**
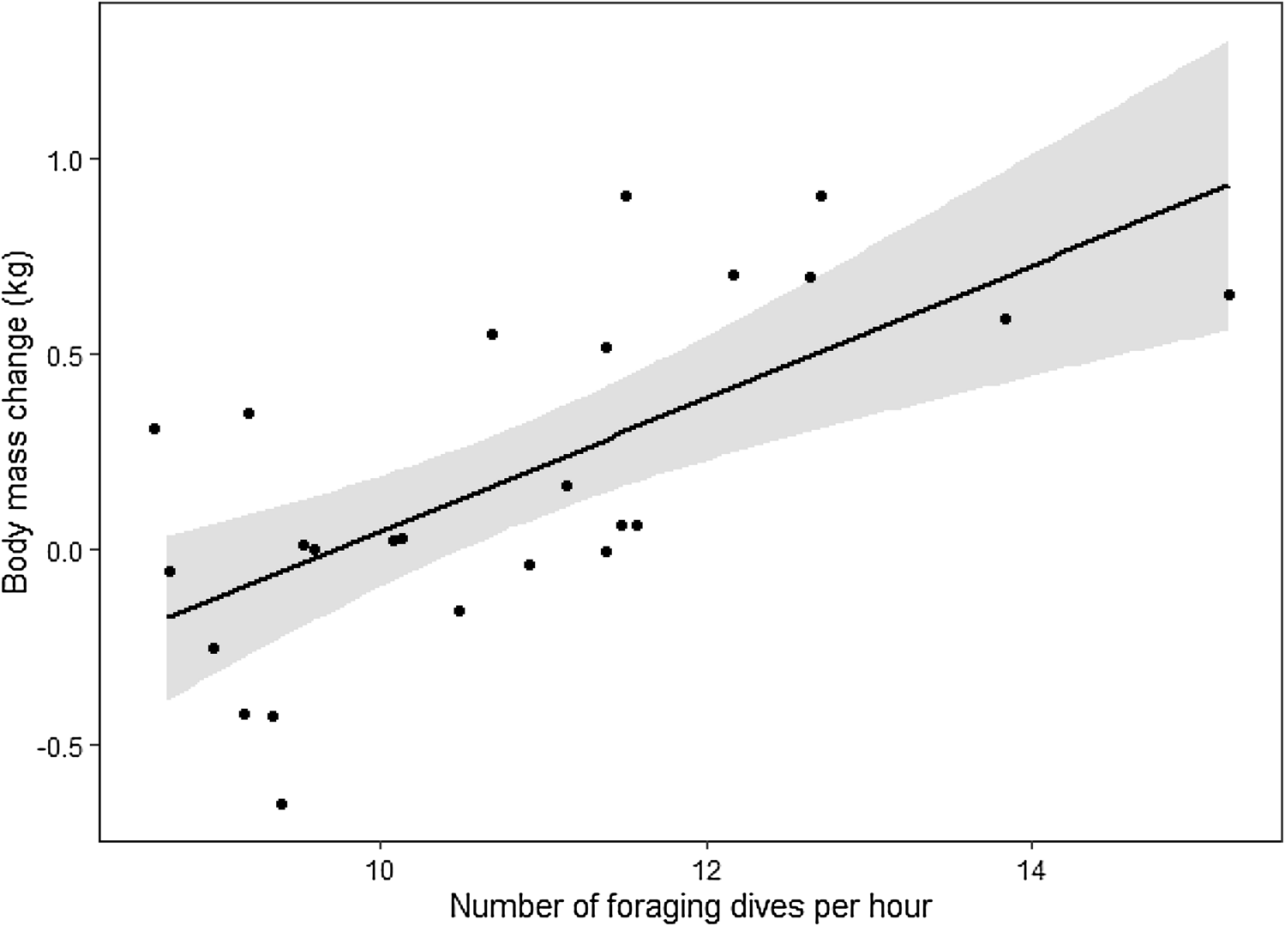
Body mass change of Adélie penguins over a foraging trip increases with the number of foraging dives they perform per hour. Linear regression equation and 95% CI from model 1: y = − 1.658 + 0.170 * Foraging_dives_per_hour ; marginal R^2^ = 0.44.

The effect of other covariates, either additive or as part of an interaction with the number of foraging dives per hour, was not strongly supported. The additive effect of study day and sex was included in 2 of the 3 top models (Table 1, models 6 and 7) but, in both models, the associated 95% CI included zero (model 6 with study day, $\widehat{\beta }$ = − 0.016, SE = 0.013, 95% CI − 0.042 to 0.011; model 7 with sex, $\widehat{\beta }$ (sex = male) =  − 0.134, SE = 0.131, 95% CI − 0.412 to 0.143). The model receiving the most support remained model 1 (Table 1), including only a linear effect of the number of dives per hour (Table 2, Fig. 4).

**Table 2:** Output of the best model fitted using restricted maximum likelihood estimation with bird ID as a random effect.

Body mass change ~ foraging dives per hour (n = 25, R^2^ = 0.44)					
Fixed effect	Value	SE	DF	t-value	p-value
Intercept	− 1.658	0.430	13	− 3.855	0.002
Foraging_dives_per_hour	0.170	0.039	13	4.325	< 0.001

R^2^ = marginal r-squared value for mixed models from Nakagawa et al.^59^.

## Discussion

The combined use of RFID technology and time-depth (GDRs) recorders allowed us to identify an easily recordable dive metric, the number of foraging dives per hour, which explained a significant part of the variation in the amount of food collected by Adélie penguins while foraging at sea. This metric can be used as an index of food intake when more direct measurements are impossible, e.g. during the non-breeding season due to the linear relationship to foraging success (in terms of body mass change over a foraging trip, i.e. not considering prey type or quality). This result supports a previous approach of using foraging dive frequency as a proxy for prey patch quality^51^ and goes further by demonstrating a direct link between foraging dive frequency and prey intake. In this previous study, the authors used the premise that “foraging dives, and especially successive ones, occur when and where food is available”. As Adélie penguins dive continuously during their foraging trips^51^, the positive relationship we found between the number of foraging dives per hour and mass gain could be used to identify areas of high feeding intensity (and potentially high prey concentration), including outside of the breeding season. While the number of undulations per hour explained a smaller proportion of the variation in foraging success than in previous studies (12% vs. 26% in^16^), the number of foraging dives per hour explained almost twice as much as the highest previous estimates.

Contrary to our initial expectations, we did not find a significant effect of sex or study day (reflecting the growing chick needs) on body mass change. It is possible that our relatively small sample size (25 foraging trips from 11 individual birds) did not allow us to detect moderate or weak effects. However, using larger sample sizes, previous studies^36,39^ found no effect of sex on meal size, and only a small effect of sex on within-trip mass change^36^, with females averaging 3.0% less mass gain per trip than males. Regarding the effect of study day, meal size has been shown to increase as the chicks were growing and entering crèches^39^ but within-trip mass change was unaffected by breeding stage^36^. We are therefore confident that sex or study day does not significantly affect body mass change or the strong relationship we detected between the number of foraging dive per hour and body mass change.

In this study, the classification of a dive into a ‘foraging’ dive (vs. ‘exploratory’ or ‘other’) does rely partly on the number of undulations as it requires a dive to reach at least 10 m in depth and exhibit ≥ 4 undulations (see methods for additional criteria). So this relatively simple metric, which only requires time-depth data and can be very easily computed, is actually a composite metric including information about the number of undulations in the dive profile, the dive depth and shape, the bottom phase duration, the dive duration and the descent/ascent rates. Considered individually, all these variables have been shown to explain some variation in foraging success in various, air-breathing marine species (see “Introduction”). Our results in Adélie penguins confirm that in species that search for and catch pelagic prey, dive type (as defined by depth, shape, duration of the different phases as well as descent and ascent rates) is an important parameter to take into account^8^. Our proposed metric to assess food intake, the number of foraging dives per hour, does combine dive type and frequency with number of undulations.

Contrary to the number of undulations alone, which may be related to the number of prey items caught (or targeted) but not to their size or energy content^4^, the number of foraging dives per hour linearly relates to the penguins’ body mass change. This mass change reflects both the mass of prey ingested and potentially part of the energy transfer between prey and predator, depending on how much digestion happened over the trip. It can therefore be used to identify foraging (and fattening) hotspots at a relatively small temporal scale at times when more direct observations or measurements are impossible (e.g. in winter or during the juvenile phase), as suggested by^51^. The residual (unexplained) variation in body mass change in our study might be partly due to the indirect effect of differences in trip durations, leading to different degrees of prey digestion among individual trips^42^. Such could blur the relationship between foraging dives and mass change, as well as differences in parental investment ‘trajectories’ over the breeding season (i.e. some parents start the breeding season in poor body condition and will invest more into self-maintenance, making longer trips and therefore digesting more before coming back to the colony to feed their chicks)^36^. This potential influence of digestion on body mass change would be attenuated when looking at hourly changes in foraging success outside of the breeding season. This index does not discriminate between prey species, which is of interest for inferring prey species’ distribution, examining individual diet preferences or interpreting different rates of chick growth^60,61^. As our study was conducted on a relatively small number of individuals and restricted period of time, replication of our experimental settings and analyses in different years and locations would be beneficial.

Future research should aim at combining an even larger array of data sources, such as quantification of the preyscape^40,62^, animal-borne video cameras^13^ and faecal DNA metabarcoding^43^, together with dive data in order to further refine dive shape classification based on the type of prey caught during a given dive. The association of specific dive shapes to a given type of prey (i.e. mainly crystal krill *Euphausia crystallorophias* or silverfish *Pleuragramma antarcticum* for the Adélie penguin in the Ross Sea) and number of prey items, as identified on video cameras and through DNA metabarcoding, would then allow us to quantify the energy gained over various periods of time.

## Supplementary Information

Supplementary Information.

## Data Availability

Data collected are available at California Avian Data Center (CADC; http://www.pointblue.org/cadc) hosted by Point Blue Conservation Science and metadata registered with the "Antarctic Master Directory" (https://www.scar.org/data-products/antarctic-master-directory/).
