# Supplementary material for: Foraging dive frequency predicts body mass gain in the Adélie penguin
Source: Sci Rep. 2021 Nov 24;11:22883. doi: 10.1038/s41598-021-02451-4 (PMC8613197; doi:10.1038/s41598-021-02451-4)
Supplement: Supplementary file 1 — Supplementary Information. [file 41598_2021_2451_MOESM1_ESM.docx]

**Body mass estimation - Details**

Penguins crossing the WB were typically on the scale platform for < 3 s, rarely standing still. Because the crossings are dynamic (i.e. bouncing from forward movement) and don’t provide a single mass measure, we developed a model to predict the body mass of the penguin based on a minimum set of records. The scale recorded 90 masses per second, all of which were stored on a 64GB microSD card. It automatically tared every 60 min to adjust for variation in snow, gravel, and other debris, and was accurate to ±30 g when compared with known static masses (tested periodically) prior to post-processing the data (described below). Similar to an approach we have used previously to calculate the most accurate penguin masses with an earlier version of the WB^1,2^, we first assigned a preliminary calculated mass to each penguin crossing (45,704 crossings total) equivalent to the mean of the top 20% of reported masses for each. We then filtered this to a subset that were realistic masses (between 2.65 kg and 5.25 kg; less would be likely to be a chick and more could represent multiple individuals on the scale^3^ and had a standard deviation of < 0.5 kg and at least 100 individual masses (meaning that the penguin was on the scale for >1 second; n = 18,758). Next, we randomly selected 20% of these crossings (n = 3751) to evaluate exploratory Boosted Regression Tree (BRT) models to use various aspects of the crossing to predict the calculated mass that had been preliminarily derived. The aspects included as covariates were: maximum mass, average mass, median mass, the number of masses (i.e., how long the penguin was on the scale), and the difference between the 10th mass recorded and a series of subsequent masses, from the 20th to the 100th, in increments of 10. We used a range of learning rates (0.01, 0.005, 0.0005) and tree complexities (1 to 5) with a maximum of 50,000 trees and selected optimal learning rate and tree complexity values based on models that minimized predictive deviance, and in case of ties, prioritized models with larger learning rates, smaller tree complexities, and fewer trees to reduce overfitting^4^ .

Based on the results of the above exploration, we then fit a BRT model predicting the preliminary calculated mass with 1600 trees (learning rate = 0.01, tree complexity = 2) and a gaussian distribution using a smaller subset of the data for which the standard deviation of the mean of the top 20% of masses was <=50g (n = 829 crossings). This resulted in a model with a mean total deviance of 0.313, mean residual deviance of 0.002, estimated cv deviance = 0.004 (SE = 0.001). Training data (75% of the data) correlation = 0.997, cross-validation correlation = 0.995 (SE = 0.001). Residuals from this model conformed to expectations of normalcy (as evaluated visually via histogram and comparison of fitted values to predicted values as well as residuals compared to fitted values) given the assumption of a gaussian distribution. Results from this model were then used to predict the best possible calculated masses for the full set of crossings. All statistics related to mass calculations were performed using the dismo package version 1.1-4^5^ for R 3.6.1^6^ with additional scripts from^4^.

**References**

1. Ballard, G., Dugger, K. M., Nur, N. & Ainley, D. G. Foraging strategies of Adélie penguins: adjusting body condition to cope with environmental variability. *Marine Ecology Progress Series* **405**, 287–302 (2010).

2. Lescroël, A. *et al.* Working less to gain more: when breeding quality relates to foraging efficiency. *Ecology* **91**, 2044–2055 (2010).

3. Ainley, D. G. *et al.* Diet and foraging effort of Adélie penguins in relation to pack-ice conditions in the southern Ross Sea. *Polar Biology* **20**, 311–319 (1998).

4. Elith, J., Leathwick, J. R. & Hastie, T. A working guide to boosted regression trees. *Journal of Animal Ecology* **77**, 802–813 (2008).

5. Hijmans, R. J., Phillips, S., Leathwick, J., Elith, J. & Hijmans, M. R. J. Package ‘dismo’. *Circles* **9**, 1–68 (2017).

6. R Core Team. *R Foundation for Statistical Computing. R: A language and environment for statistical computing*. (2019).
